# Early Specific Host Response Associated with Starting Effective Tuberculosis Treatment in an Infection Controlled Placebo Controlled Mouse Study

**DOI:** 10.1371/journal.pone.0057997

**Published:** 2013-02-28

**Authors:** Alice L. den Hertog, Alex F. de Vos, Paul R. Klatser, Richard M. Anthony

**Affiliations:** 1 Royal Tropical Institute, KIT Biomedical Research, Amsterdam, The Netherlands; 2 Center for Experimental and Molecular Medicine, Center for Infection and Immunity Amsterdam, Academic Medical Center, Amsterdam, The Netherlands; Johns Hopkins University School of Medicine, United States of America

## Abstract

Recently we proposed exploring the potential of treatment stimulated testing as diagnostic method for tuberculosis (TB). An infection controlled placebo controlled mouse study was performed to investigate whether serum cytokine levels changed measurably during the early phase of TB chemotherapy. Serum was collected prior to and during the first 3 weeks of isoniazid (INH) and rifampicin (RIF) chemotherapy, and levels of 23 selected cytokines/chemokines were measured using a liquid bead array. The serum levels of IFNγ, IP-10, MIG, MCP-1, IL-17 and IL-6 were elevated in the TB infected mice compared to non-infected mice at least at 1 time point measured. In infected mice, IFNγ, IP-10, MIG and MCP-1 levels decreased within 7 days of treatment with RIF+INH compared to placebo. Treatment of non-infected mice in the absence of tuberculosis infection had no effect on these cytokines. IL-17 and IL-6 had decreased to baseline in all infected mice prior to the initiation of treatment. This study demonstrates that systemic levels of some cytokines, more specifically IFNγ, IP-10, MIG and MCP-1, rapidly and specifically change upon starting TB chemotherapy only in the presence of infection in a mouse model. Thus, IFNγ, IP-10, MIG and MCP-1 are promising ‘Treat-to-Test’ targets for the diagnosis of TB and deserve further investigation in a study on human TB suspects.

## Introduction

Tuberculosis (TB) diagnosis remains challenging, especially in low income countries where molecular tools and culture are often not available or difficult to widely apply. There are no useful immunodiagnostics to diagnose TB disease [1]. Tuberculin skin tests (TST) and IFNγ release assays (IGRA) are used for detection of a TB-specific host response, but these assays are not recommended for the detection of active disease [2]. At the other side of the immunological spectrum, the humoral immune response to TB is very variable and no accurate diagnosis can be made based on the presence or levels of antibodies against any target [3].

In view of these intrinsic difficulties, and the lack of progress towards a true simple near patient diagnostic, we recently proposed to research the feasibility of a Treat-to-Test strategy, a pragmatic alternative to single point testing [4]. This strategy would consist of prescribing a few doses of TB drugs to a suspect, and measuring the bacteriological or immunological response to this intervention. In patients not showing a response this approach should be backed up by more extensive testing, for (drug resistant) tuberculosis or another differential diagnosis.

It has been demonstrated that TB chemotherapy affects cytokine/chemokine profiles. Djoba Siawaya *et al.*(2009) [5] have shown that in plasma of TB patients the levels of a number of cytokines (amongst others IP-10, MCP-1, MIP-1α, Eotaxin) change within 1 week's chemotherapy. IP-10 and VEGF plasma were shown to decrease in TB patients with and without HIV after initiation of anti-tuberculosis chemotherapy [6]. Furthermore, in a recent mouse study it was established that successful therapy caused a decrease in plasma levels of IFNγ, IL-17, IP-10 and MIG [7] within 5 weeks. Although these studies show that therapy induces - sometimes rapid - changes in immunological status, it cannot be excluded that these effects are due to TB-unrelated effects of the antimicrobials, possibly due to disruption of normal bacterial flora or side effects (eg. hepatoxicity), and thus may occur also in the absence of *M. tuberculosis* infection.

An argument for a rapid host response after starting TB chemotherapy in infected individuals is the demonstrated rapid reduction of viable bacteria. Early bactericidal activity (EBA) studies have demonstrated that the majority of the bacilli within the lungs are killed by INH within a few days [8], [9]. Our hypothesis is that there will be an immunological response to bacterial degradation measurable as an increase or decrease in specific immunological markers within a short period after taking the TB drugs.

Therefore, we designed an infection controlled, placebo controlled study in a murine pulmonary TB model. Mice were experimentally infected with TB (or not infected in control groups) for 6 weeks and then started on RIF+INH (or placebo) therapy. Serum was collected 2 weeks before and 0, 3, 7 and 21 days after start of therapy. Thus, we could investigate the effects of the killing of tuberculosis by the drugs, and exclude any effects of the drugs directly on the host or through effects on host microflora.

## Results

### Disease controls

**Figure 1:**
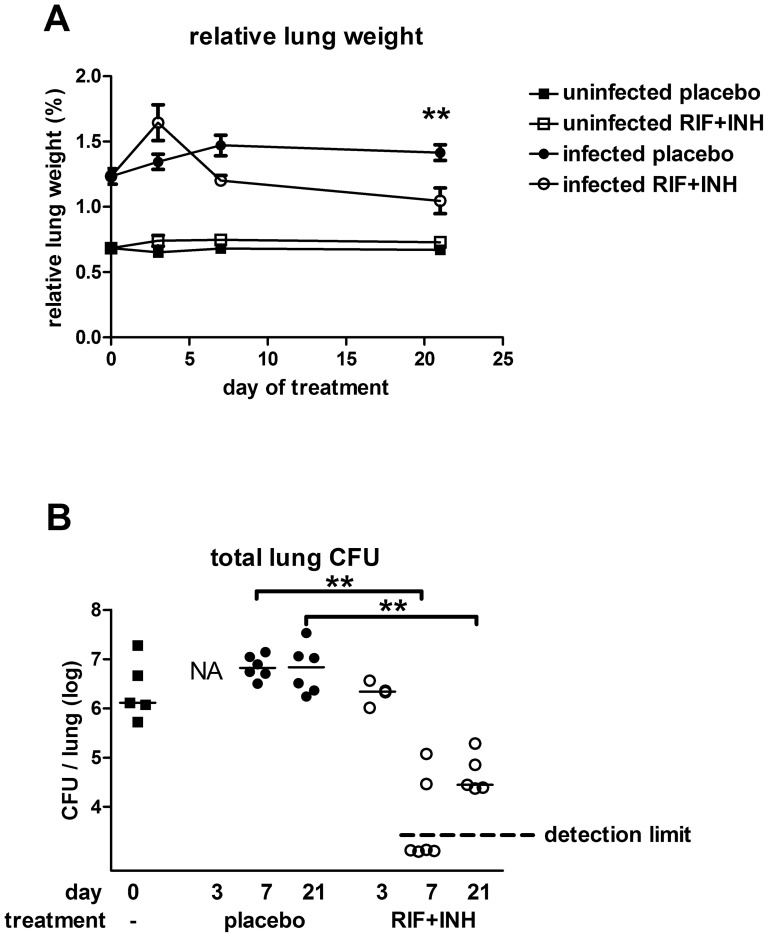
Lung CFU and relative lung weights of mice. **A**: Relative lung weights were calculated as a percentage of total body mass (mean +− SE). Black squares: uninfected placebo, open squares: uninfected RIF+INH treated, black circles: infected placebo, open circles: infected RIF+INH treated mice. At start of treatment, relative lung weights were increased in infected mice compared to uninfected mice (p<0.001). After an initial increase within 3 days, relative lung weight started to decrease within 3–7 days after start of treatment with RIF+INH in the infected mice although remaining higher than in the uninfected mice until the end of the experiment (p<0.01). Treatment had no effect on lung weight in uninfected mice. **B**: CFU counts of lung homogenates of sacrificed mice. Median baseline level in infected mice prior to treatment was 1.3×10^6^CFU per lung (black squares) and similar levels were found after 7 and 21 days of placebo treatment (black circles, 3 days not available due to contamination of culture plates). Treatment with RIF+INH strongly reduced CFU counts within 7 days (open circles; detection limit 2×10^3^ CFU/lung). Counts from 1 mouse are not available due to contamination. Cultures of undiluted lung homogenates from uninfected mice yielded no TB colonies (not shown). ** p<0.01

To confirm successful infection, TB lung counts of 2 mice sacrificed 1 day after the infection were performed, yielding ∼180 CFU/lung. Furthermore, levels around 10^6^ to 10^7^ TB CFU per lung as well as an increase in lung weight relative to body weight (‘relative lung weight’) were detected in all infected mice sacrificed before treatment and in placebo groups (p<0.0001; Figure 1).

Treatment with INH and RIF did not affect the relative lung weight in uninfected mice. In infected mice, the relative lung weight increased within 3 days after starting treatment in the infected mice (p<0.01) and strongly decreased between 3–7 days after starting treatment to below that of the placebo treated infected mice. Relative lung weight further decreased in treated mice until the end of the experiment, 3 weeks after start of therapy, although remaining higher than the lung weight of uninfected mice (p<0.01; Figure 1A).

Lung CFU counts of *M. tuberculosis* were also obtained from all sacrificed mice before and during the course of treatment. The lung CFU of infected mice in the placebo groups remained at a stable level (Figure 1B), whereas in the treated groups a 100 fold decrease in CFU/lung was seen between 3 and 7 days (Figure 1B). The lung cultures of uninfected mice yielded no TB colonies.

When the volumes collected allowed, 50 µl of each serum and urine sample was cultured undiluted to screen for the presence of TB. However, no TB CFU were detected at any time point in any of the 39 sera or 18 urine samples of infected mice tested (data not shown).

### Cytokine levels prior to the initiation of treatment

The whole panel of immunological markers was screened for 2–4 mice per group. Of the cytokines/chemokines studied only IFNγ, IP-10, MIG, MCP-1, IL-17 and IL-6 showed measurable shifts in concentration that could be related to infection and or treatment (Figure 1 and File S1). In a second round of testing, levels of these 6 cytokines + IL-1a and MIP-1α were tested in all samples and their kinetics studied in detail. Although KC median levels were higher in infected than uninfected groups in the 1^st^ round of testing, they did not appear to differ between RIF+INH and placebo treated groups and thus it was decided not to include this biomarker in the 2^nd^ round of testing (File S1).

**Figure 2:**
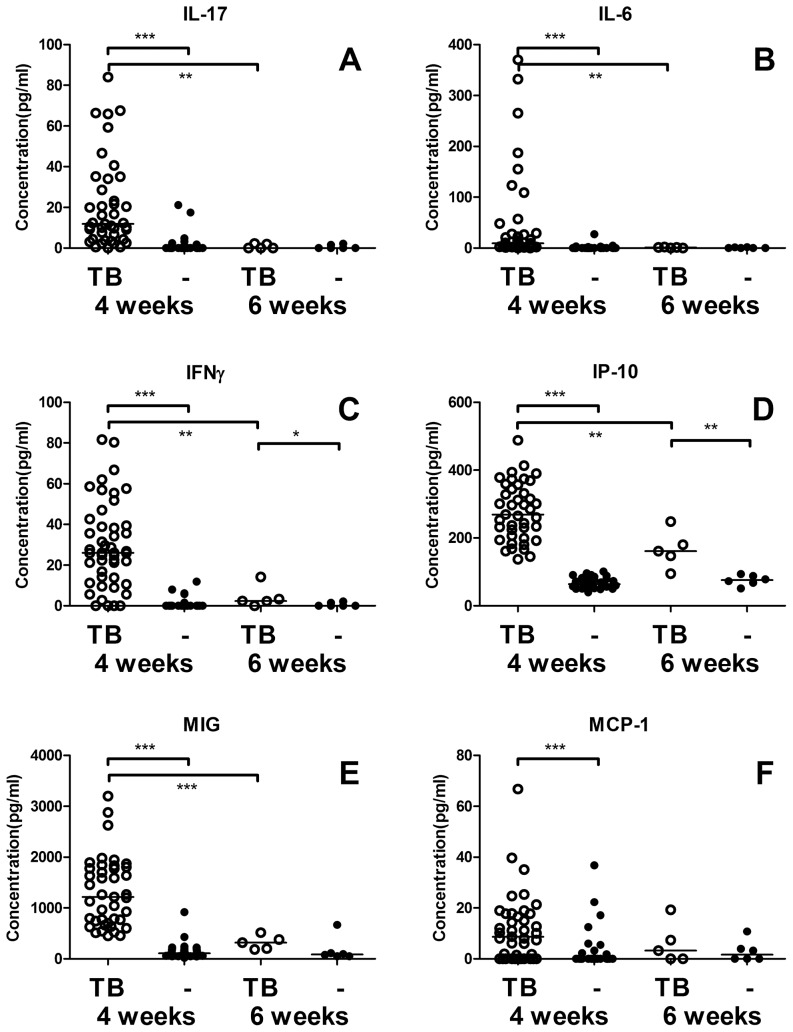
TB associated cytokines after 4 and 6 weeks of infection. IL-17 (**A**) and IL-6 (**B**) are significantly increased in the infected mice (open symbols) compared with uninfected mice (black symbols) at 4 weeks after infection. At 6 weeks post infection levels in all infected and non-infected mice were similar and around the detection limit for both cytokines. IFNγ (**C**), IP-10 (**D**)and MIG (**E**) are increased both at 4 and 6 weeks after infection compared to non-infected mice. As MIG levels were higher in all infected mice than in all uninfected mice except 1, at 6 weeks significance was not reached (p = 0.08). Levels of MCP-1 (**F**) were increased in infected mice at 4 weeks post infection compared to uninfected mice. At both 4 and 6 weeks post infection the range of MCP-1 levels was quite wide between both infected and uninfected mice and levels at 6 weeks were very similar between infected and uninfected mice. * p<0.05, ** p<0.01, *** p<0.001

At 4 weeks post infection, IFNγ, IP-10, MIG, MCP-1, IL-17 and IL-6 were elevated in infected mice compared to the uninfected mice. However, before the start of treatment (6 weeks after infection) IL-17 and IL-6 levels were below 2.2 pg/ml (or undetectable) in all samples (Figure 2A, 2B). IFNγ, IP-10 and MIG levels also showed a significant decrease in levels between 4 and 6 weeks although IFNγ and IP-10 remained significantly increased compared to the uninfected groups (Figure 2C-E). At 6 weeks, MIG levels were higher in all infected mice than in all uninfected mice except 1 (p = 0.08). The levels of MCP-1 varied greatly between mice (both uninfected and infected) and were similar between both groups at 6 weeks post infection (Figure 2F).

### Cytokine levels during RIF+INH treatment/placebo

At 6 weeks post infection treatment with RIF+INH or placebo was started. Samples collected at 3, 7 and 21 days post infection were analysed to determine whether treatment affected cytokine levels.

**Figure 3:**
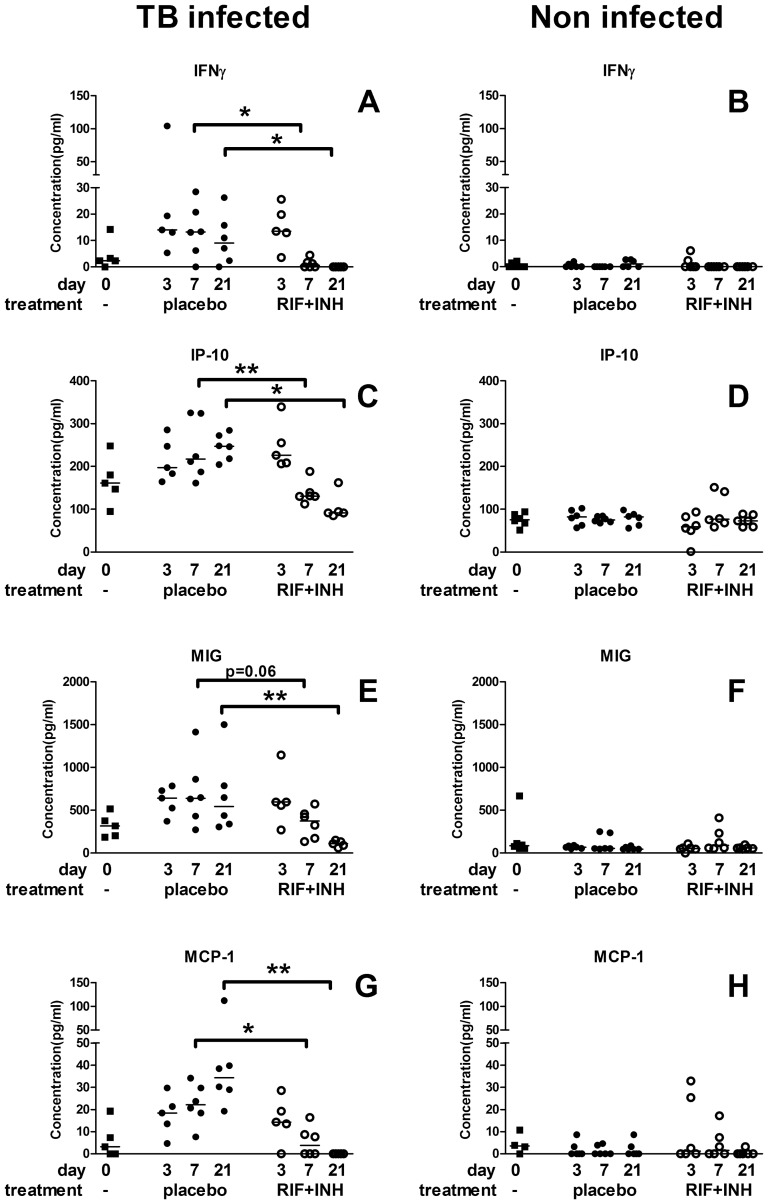
Changes in cytokine levels during TB treatment. IFNγ, IP-10, MIG and MCP-1 levels were measured in infected (left hand panels, A, C, E, G) and non-infected (right hand panels, B, D, F, H) mice before (black squares) and after 3, 7, and 21 days of treatment with RIF+INH (open circles) or placebo (black circles). In the infected groups within 7–21 days of treatment with RIF+INH levels of all 4 cytokines were lower compared to placebo treated mice. In the non-infected mice no differences in levels were seen between different treatments or time points. * p<0.05, ** p<0.01, *** p<0.001

After 7 days, levels of IFNγ, IP-10 and MCP-1 were significantly decreased in the treated versus placebo groups of infected mice (Figure 3A, 3C, 3E). After 21 days, a significant difference was also found for MIG (Figure 3D). No effect of treatment on any of these four cytokines was detected in uninfected mice (Figure 3B, 3D, 3F, 3H). At 21 days after starting treatment, the levels of all 4 cytokines in the infected treated mice were similar to those of both groups of uninfected mice at that time point (Figure 3).

The other cytokines from the 23-plex panel were just above or below the detection limit in the majority of the samples, or did not show a correlation with TB infection or treatment (File S1).

## Discussion

Serum samples were collected from an infection-controlled, placebo-controlled murine model for pulmonary TB during the early phase of therapy and tested with a 23-plex cytokine panel. The 23 cytokines measured were selected based on any previously published clinical data on serum levels during TB infection, disease, treatment or BCG vaccination in human or mouse studies or upon stimulation of blood with TB antigens (described amongst others by Mattos *et al.* 2010, Djoba Siawaya et al. 2009, Pokkali *et al.* 2009, Smith *et al.* 2010 [5], [10]–[12]). Of the 23 analytes measured in this study, all but 6 (IFNγ, IP-10, MIG, MCP-1, IL-17 and IL-6) showed no discernible association with 4–9 weeks infection or with chemotherapy.

Four (IFNγ, IP-10, MIG and MCP-1) of these 6 cytokines responded to therapy, all of which have been previously associated with TB disease [6], [7], [13]. IP-10 and MIG are both induced by IFNγ, which is a key molecule in the Type 1 response in human tuberculosis and is used as readout in IGRA assays. It has been shown that in IGRA assays, not only IFNγ, but also both IP-10 and MIG levels are increased in stimulated blood from latently infected tuberculosis patients [14], [15]. The association between TB disease and MCP-1 is less well established, as in some studies a positive correlation [16], [17] was seen but in others there was a negative correlation [13]. However, as with IFNγ, IP-10 and MIG it has been shown that MCP-1 release is increased when stimulating whole blood of TB patients with TB antigens [14].

The remaining 2 cytokines (IL-17 and IL-6) showed only an association with infection. IL-17 serum levels were increased at 4 weeks post infection compared to controls as previously reported [7]. However de Steenwinkel *et al.* (2012) [7] found that levels in treated mice were lower than in placebo treated mice after five weeks of therapy whereas in our study IL-17 and IL-6 levels in infected mice had already decreased to baseline before therapy was initialized (6 weeks after infection; Figure 2). The early IL-17 response corresponds to the model proposed by Torrado and Cooper (2010) [18] in which IL-17 is stimulated upon primary infection, but counterbalanced by TH1 responses in later stages of disease.

Our finding that within 1 week of treatment systemic levels of 4 cytokines show a decrease within a concentration range measurable by ELISA is very promising for the TB Treat-to-Test approach. Indeed, effects of tuberculosis treatment on serum cytokine levels have been observed previously in humans [5], but the added value of the present study is the availability of controls both for infection and treatment, which suggests these effects are linked to the killing of mycobacteria. The data presented here provides valuable background for clinical studies in humans.

Using this setup we were able to detect immunological changes due to the combined antimycobacterial action of RIF and INH, whilst controlling for effects unrelated to the killing of *M. tuberculosis*. The activity of INH is quite specific for TB as it is a pro-drug that requires activation by *M. tuberculosis* KatG and killing of host microflora or other infectious agents is probably limited. RIF has a broader spectrum of activity [19] and has also been shown to reduce human microflora after only a few doses [20], thus a change in immunological response as a result of these effects cannot be excluded. Direct effects of the drugs on the host, e.g. hepatotoxicity, which is a major side effect of both drugs, could also induce immunological changes reflected in altered serum levels of certain cytokines/chemokines. Therefore, it is reassuring that in this mouse study we identified a number of markers whose serum levels were rapidly affected by therapy in infected mice but were stable in non-infected mice treated with the same antibiotics. Nonetheless it must be realized this is only a preliminary study and the non-infected mice in this study are not comparable to human suspects who may have pulmonary complaints or other infectious diseases that may give a response to RIF+INH treatment.

A limitation of our study is that as blood volume in mice is small, limiting repeated sampling of sufficient volume from individual animals. Therefore we compared the levels between groups of RIF+INH and placebo treated mice instead of using serial samples from individual mice at all time points. We expect that repeated sampling would have increased the power of our experiment as individual variations would have been be minimized. Indeed, if used diagnostically to demonstrate TB infection two serial samples would be tested; one collected before and one collected at a specific time point early after starting therapy. However, of course the variation between human TB patients will be much larger than between the mice in this model in respect of amongst others disease stage, infecting strain, and comorbidities. Also, in this study the mice received the two drugs INH and RIF which are responsible for the majority of the early bacteriological effect of the current standard first line tuberculosis chemotherapy [8], [9] but TB patients usually also receive additional drugs for example pyrazinamide and ethambutol. Clinical studies are necessary to investigate the effects of these factors in TB suspects.

As an immunocompromised state as a result of HIV infection is one of the most important risk factors for TB, it is worth noting that MCP-1 and IP-10 are likely to behave similarly upon treatment in HIV infected and uninfected TB patients: IP-10 and MCP-1 have been shown to be elevated in TB both with and without HIV infection (the levels of MCP-1 were even more increased in HIV+ TB patients) and a decrease in serum levels in TB patients receiving treatment was shown for both HIV- and HIV+[6], [17].

In addition to cytokines, antibody levels may also change dramatically upon starting treatment although antibody responses of individual patients are known to be very diverse [3], in fact probably much more diverse than cytokine responses. Nonetheless antibody titers to a number of TB antigens have been shown to increase shortly after initiation of effective therapy [21] and antibody profiles tend to shift towards intracellular epitopes during therapy [10]. As the immune system of a TB infected individual will have been primed during the period of infection before becoming symptomatic, the release of antigens upon bacterial killing may cause a rapid boost in antibody production. Thus antibodies also remain interesting candidates to be explored for a Treat-to-Test approach.

It could be argued that a Treat-to-Test approach may encourage the overuse of antimycobacterial drugs. But in many settings, the lack of available diagnostic methods for TB forces clinicians to decide whether to treat TB suspects empirically, with only a clinical diagnosis or give broad spectrum antibiotics to rule out pulmonary infection due to other bacterial species. This leads to treatment delay in some TB patients and possibly overtreatment as patients without bacteriologically confirmed disease receive a full course of DOTS [22] which could be avoided with a Treat-to-Test approach.

As the Treat-to-Test approach is effectively a form of treatment monitoring, it is evident a diagnosis will only be confirmed when treatment is given to which the infectious agents are susceptible. However, this does not mean that such an approach would by definition be useless in a population with high (M)DR rates. As noted above, first line therapy may often be prescribed empirically without bacteriological diagnosis. Thus a Treat-to-Test approach could aid in identifying patients responding to the given drug(s), and rapidly detect suspects not responding, either due to drug resistance or incorrect diagnosis. Both these nonresponders would need further investigations which may include preferential access to molecular tests such as GeneXpert to determine whether they have (M)DR TB, or another disease. Such a strategy could significantly improve the cost effectiveness and the willingness to pay for very effective but more complex assays that would otherwise be difficult to justify [23].

Here we demonstrate that serum levels of a number of immunological markers change measurably within 1 week's anti tuberculosis therapy in a mouse model for TB only in infected mice. This supports the potential of a Treat-to-Test approach for TB diagnosis. Clinical studies are necessary to validate these results in human TB suspects and to determine whether this approach will have utility in TB diagnosis. Treatment monitoring and detection of drug resistance could also be facilitated by knowledge of the kinetics of markers for bacteriological response. Collection and testing of samples in the early stages of chemotherapy suitable to study the kinetics of easily measurable and potentially highly informative markers is a priority.

## Materials and Methods

### Mice

Female Balb/c mice were provided by Charles River (France) and kept in specific pathogen free housing in the AMC animal facility.

The research described in this paper complied with the ethics guidelines of the University of Amsterdam. All experiments were approved by the Animal Care and Use Committee of the University of Amsterdam (Project Number: 102123AA).

### Infection model

Six week old mice were infected intranasally with approx. 325 CFU of *M. tuberculosis* Erdman strain in 50 µl of a sterile 0.9% NaCl solution. The infectious load was confirmed by plating an aliquot of the inoculum and successful infection was confirmed by lung counts of 2 mice sacrificed 1 day after infection. Control mice received vehicle (50 µl sterile 0.9% NaCl solution) only.

### Tuberculosis model and chemotherapy

To both infected and uninfected mice, therapy was administered from 6 weeks after the infection until sacrifice 3, 7 or 21 days later. The regimen consisted of a combination of rifampicin (10 mg/kg) and isoniazid (25 mg/kg) administered 5 days per week by gavage (in 200 µl 5% sucrose in water). Antibiotics were prepared by dissolving RIF (Sigma-Aldrich Chemie B.V., Zwijndrecht, The Netherlands) at 200 mg/ml DMSO, and further dilution to 1 mg/ml in sucrose buffer to improve palatability, after which 25 mg/ml INH (Sigma-Aldrich) was added. As treatment control, groups of infected and uninfected mice were treated similarly with 0.5% DMSO in 5% sucrose solution (placebo).

### Lung counts

At specific time points, mice were sacrificed and weighed, and lungs were isolated aseptically and weighed. After the addition of 4 volumes of sterile 0.9% NaCl solution the lungs were homogenized at 4°C with a tissue homogenizer (Biospect Products, Bartlesville, OK, USA). Serial 10-fold dilutions of the homogenates were made in sterile 0.9% NaCl solution of which 50 µl were plated onto Middlebrook 7H11 agar plates. Lung samples from TB infected mice were plated with additional 10^−2^, 10^−3^ and 10^−4^ dilution, for non-infected mouse samples were also plated undiluted and with 10^−1^ dilution. Colonies were counted after 3–4 weeks incubation at 37°C. The CFU are reported per lung, rather than CFU per gram of lung tissue, to account for differences in the weight of the lungs due to individual variation and inflammatory processes. The limit of detection for each dilution series was calculated based on the lowest dilution plated and initial weight of the lungs, if no bacteria were cultured from the infected mice the limit of detection was plotted (Figure 1B).

### Sample collection

Baseline serum samples of all mice were collected 2 weeks prior to start of treatment (4 weeks after infection) by submandibular vein puncture. Treatment or placebo was started 6 weeks after infection, and serum samples were collected after 0, 3, 7 and 21 days of treatment. At these time points, all mice (n = 5–6) from each group (infected/uninfected; RIF+INH treatment/placebo) were sacrificed and blood was collected by heart puncture. After clotting, and centrifugation, serum samples were stored at −70°C until use. Four TB infected mice were lost prior to treatment with antibiotics or placebo, reducing the number of samples to n = 5 at day 0, at days 3 of placebo or RIF+INH treatment and at day 21 of RIF+INH treatment.

### Cytokine/chemokine assays

Cytokine/chemokine levels in serum samples were measured using xMAP technology (Luminex, Austin, Texas, USA). A subset of 2–4 samples per group at all time points were tested with a mouse 23-plex cytokine/chemokine analyte panel (Millipore B.V., Amsterdam, The Netherlands) consisting of Eotaxin, GM-CSF, M-CSF, IFNγ, IL-1α, IL-1β, IL-2, IL-4, IL-6, IL-7, IL-12(p40), IL-12(p70), IL-15, IL-17, IP-10, MIP-1α, MIP-1β MIP-2, KC, MCP-1, MIG, RANTES and TNFα. These markers were selected on the basis that they are measurable using an ELISA assay and have a previously reported association with tuberculosis disease [5], [10]–[12]. Based on the results of this first round of testing, the remaining samples were tested for the most promising candidate markers IFNγ, IP-10, MIG, MCP-1, IL-17, IL-6, IL-1α and MIP-1α.

Tests were performed adhering to the manufacturer's protocol using undiluted samples in duplicate if sample volume allowed. This was the case for all samples (except 1 sample that was 2-fold diluted) collected at time points 0 to 3 weeks after start of treatment/placebo. When this was not possible, samples were diluted up to 3 times. From the submandibular vein blood samples taken 2 weeks prior to the start of treatment (or placebo), 33 samples could only be included in monoplo due to limited sample volumes. Data analysis was performed using Milliplex analyst version 3.5.5.0 software (Vigenetech, Carlisle, Massachusetts, USA, www.vigenetech.com).

### Statistical analysis

Statistical analysis was performed using GraphPad Prism version 5.03 for Windows (GraphPad Software, San Diego California USA, www.graphpad.com). Analysis of relative lung weights was performed using 2-tailed parametrical T test. Total lung CFUs and all cytokine data was tested non-parametrically, using a 2-tailed Mann-Whitney test.

## Supporting Information

File S1 **Cytokine levels measured from all serum samples.** All cytokine levels measured in pg/ml for are shown. Empty cells indicate a specific marker was not measured in that sample.
(JPG)
