# Supplementary figures and images for: Early Specific Host Response Associated with Starting Effective Tuberculosis Treatment in an Infection Controlled Placebo Controlled Mouse Study
Source: PLoS One. 2013 Feb 28;8(2):e57997. doi: 10.1371/journal.pone.0057997 (PMC3585278; doi:10.1371/journal.pone.0057997)

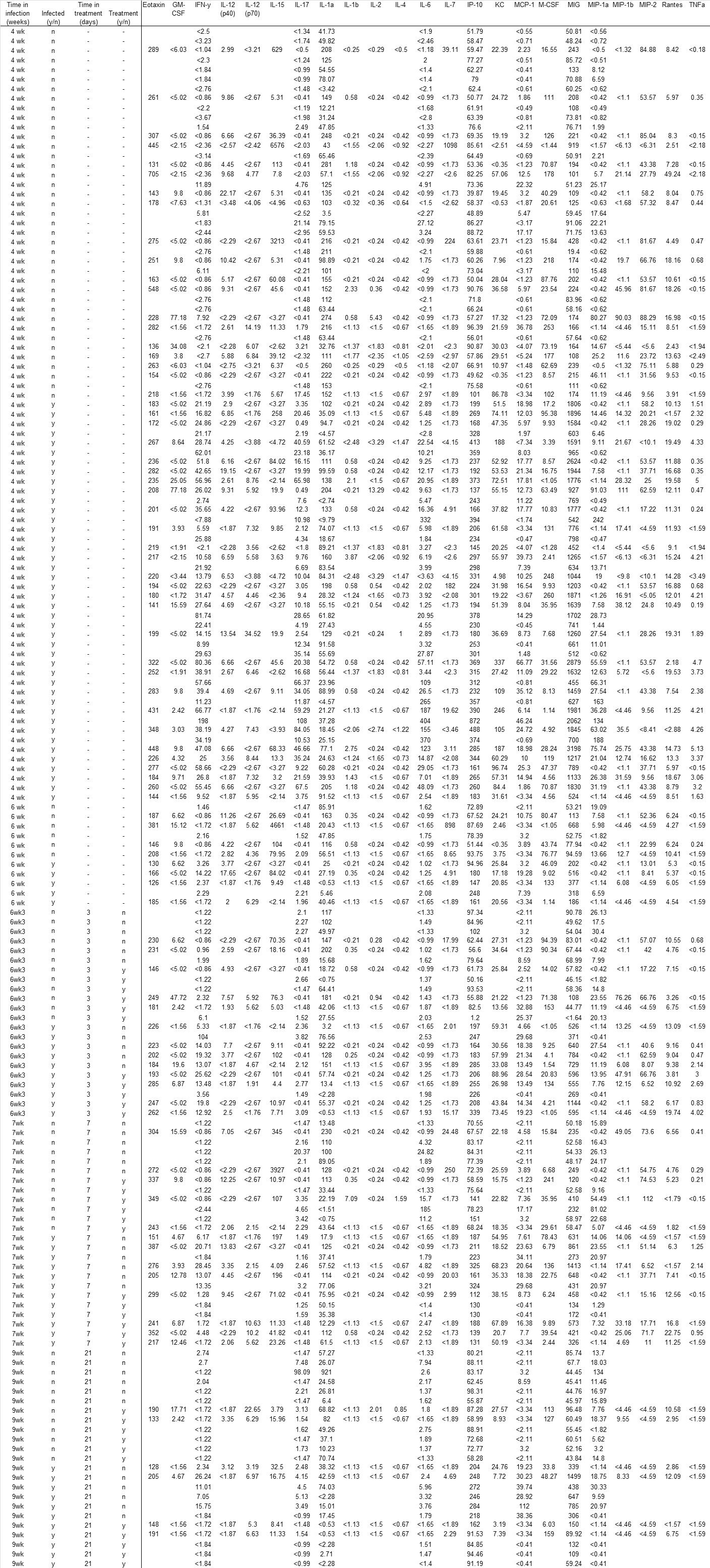

Supplement: File S1 — Cytokine levels measured from all serum samples. All cytokine levels measured in pg/ml for are shown. Empty cells indicate a specific marker was not measured in that sample. (JPG) [file pone.0057997.s001.jpg]
